# Host Gasdermin D restrains systemic endotoxemia by capturing Proteobacteria in the colon of high-fat diet-feeding mice

**DOI:** 10.1080/19490976.2021.1946369

**Published:** 2021-07-18

**Authors:** Yujie Shi, Yixin Zou, Yonghong Xiong, Shiyao Zhang, Mingming Song, Xiaofei An, Chang Liu, Wenxiang Zhang, Siyu Chen

**Affiliations:** a State Key Laboratory of Natural Medicines, China Pharmaceutical University, Nanjing, China; b School of Life Science and Technology, China Pharmaceutical University, Nanjing, China; c Department of Endocrinology, Affiliated Hospital of Nanjing University of Chinese Medicine, Nanjing, China

**Keywords:** Gasdermin D, metabolic endotoxemia, *Proteobacteria*, Cardiolipin, host and microbiota crosstalk

## Abstract

Gasdermin D (GSDMD) functions as a key pyroptotic executor through its secreted N-terminal domain (GSDMD-N). However, the functional relevance and mechanistic basis of the precise roles of host colonic GSDMD in high-fat diet (HFD)-induced gut dysbiosis and systemic endotoxemia remain elusive. In this study, we demonstrate that HFD feeding triggers GSDMD-N secretion of both T-lymphocytes and enterocytes in mouse colons. GSDMD deficiency aggravates HFD-induced systemic endotoxemia, gut barrier impairment, and colonic inflammation. More importantly, active GSDMD-N kills the *Proteobacteria* phylum via directly interacting with Cardiolipin. Mechanistically, we identify that the Glu236 (a known residue for GSDMD protein cleavage) is a *bona fide* important site for the bacterial recognition of GSDMD. Collectively, our findings explain the mechanism by which colonic GSDMD-N maintains low levels of HFD-induced metabolic endotoxemia. A GSDMD-N mimetic containing an exposed Glu236 site could be an attractive strategy for the treatment of HFD-induced metabolic endotoxemia.

## Introduction

Gut dysbiosis has been demonstrated to be associated with multiple human diseases, including diet-induced obesity.^1^ However, the underlying mechanisms driving this dysbiosis and the crosstalk between host cells and gut microbiota remain elusive. Among the gut flora, a bloom of *Proteobacteria* is the most consistent and robustly corresponded to the ecological changes during the gut dysbiosis.^2^ *Proteobacteria* is a minor component existed in a balanced environment of gut microbiota.^3^ Unfortunately, chemicals and pathogen infections are known to trigger intestinal inflammation, and thus increasing the abundance of *Proteobacteria* in the mouse models.^4^ Similarly, such an uncontrolled expansion of *Proteobacteria* is also observed in humans suffered from either severe or mild intestinal inflammation, including inflammatory bowel disease (IBD),^5^ colorectal cancer^6^and metabolic syndrome.^3^ Pathologically, as gram-negative bacteria, *Proteobacteria* inherently function as producers of pro-inflammatory factors since their abilities in lipopolysaccharide (LPS) production.^7^ This characteristic triggers the immune response in the intestines and further the systemic endotoxemia.^8^

Notably, systemic and chronic low increases in gut-derived endotoxin (LPS) have been demonstrated to facilitate the exacerbation of pathogenic metabolic perturbations, such as HFD-induced obesity.^9^ HFD feeding unfavorably increases the abundance of *Proteobacteria* and thus damages the intestinal epithelial barrier.^10^ Moreover, detrimental serum LPS, which is elevated by such damage, further disrupts the intestinal epithelial barrier and increases peripheral endocannabinoid tone, thus forming a harmful positive feedback loop.^11^ This pathological progression is termed metabolic endotoxemia and may progress to metabolic syndrome. Nevertheless, dysfunction of the intestinal barrier exposes immune/inflammatory cells to excessive amounts of LPS and bacteria, triggering an inflammatory state that results from their strong response to bacteria-initiated molecular events and is accompanied by subsequent accumulation of proinflammatory cytokines.^12^ More importantly, increased numbers of CD4-positive T lymphocytes accompany the elevated abundance of *Proteobacteria* in the context of western diet feeding, indicating remarkable correlations among colonic epithelial cells, immune/inflammatory cells, and the gut microbiota.^13^ However, the crosstalk among these populations that is involved in HFD feeding-induced low-grade metabolic endotoxemia remains elusive.

LPS produced by gram-negative bacteria is a primary activator of systemic inflammation and acts in part by increasing the activities of inflammasomes, which are composed of nucleotide-binding oligomerization domain protein-like receptor proteins (NLRPs), apoptosis-associated speck-like protein (ASC) and proinflammatory caspases (caspase-1 and caspase-11).^14^ These inflammasomes regulate the activities of caspases and govern the cleavage of the pro-interleukin-1β (IL-1β) and pro-IL-18 precursors into their mature forms, which induce a new type of programmed cell death named pyroptosis.^15^ Gasdermin D (GSDMD) is a generic substrate of inflammatory caspases and functions as a key executor of pyroptosis through the release of cleaved GSDMD-N and controlling the release of IL-1β.^16^ Of note, the organ-specific roles of GSDMD in maintaining metabolic and inflammatory homeostasis have been well documented in recent years. For instance, liver-specific GSDMD-N drives cell pyroptosis and activates the processes of diet-induced hepatic steatosis and steatohepatitis in mice.^17^ Consistently, inhibition of adipose-specific GSDMD by melatonin alleviates the obesity-induced systemic inflammatory response.^18^ Hence, these findings strongly suggest that the loss of GSDMD function represents a new approach for the treatment of metabolic disorders. In contrast, immune-specific GSDMD contributes to removing pathogen-infected intestinal epithelial cells and inhibits dextran sulfate sodium (DSS)-induced colitis in mice.^19^ In addition, GSDMD also serves as an antibacterial peptide to protect organisms from excessive infiltration of exogenous gram-negative bacteria, including *E. coli, S. typhimurium*, and *B. cenocepacia*.^16,20,21^ It should be noted that the human colon contains the most abundant and diverse assemblage of endogenous bacteria, which are disrupted in response to HFD feeding. However, the reciprocal relationship between colonic GSDMD and the endogenous gut microbiota is still unknown. The aim of this study was to identify the actual role of GSDMD in the diet-induced disruption of gut health and related diseases.

## Results

### HFD triggers GSDMD-mediated pyroptosis in the mouse colon

**Figure 1.**
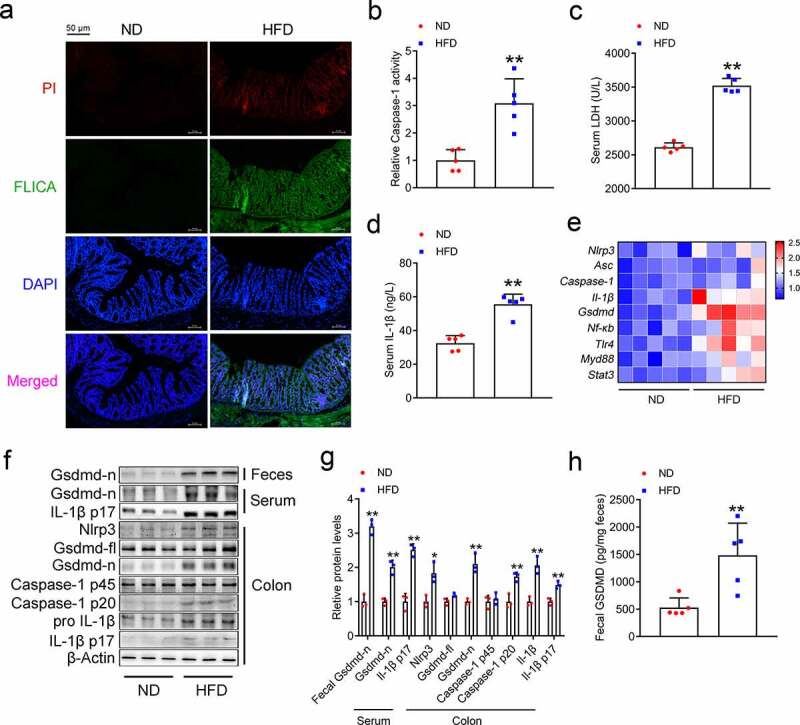
HFD Triggers GSDMD-mediated Pyroptosis in the Mouse Colon

To investigate the role of pyroptosis in the colons of mice fed a HFD, we first evaluated the activity of Caspase-1, a key upstream factor involved in the pyroptotic process, by using FLICA and PI staining analysis. Compared to the normal diet (ND), the HFD increased the immune florescence signals of both Caspase-1 and PI in the mouse colons (Figure 1(a)), and this finding was further confirmed by analyzing the colonic Caspase-1 activity (Figure 1(b)). Serologically, the serum levels of LDH and IL-1β were significantly increased in the HFD-fed mice (Figure 1(c,d)). At the mRNA and protein levels, canonical pyroptotic proteins were induced by HFD feeding, notably active GSDMD (GSDMD-N), cleaved-Caspase-1 and mature IL-1β were increased in the colons of these mice (Figure 1(e–g)). Since GSDMD-N is secreted, we next examined the serum and fecal levels of active GSDMD and found that they were consistently upregulated by HFD feeding (Figure 1(f,h)). These results indicate that GSDMD-mediated pyroptosis plays a role in the colons of HFD-fed mice.

10-week-old mice were fed either a HFD or a normal diet for 16 weeks. (a) IF analysis of propidium iodide (PI) and FLICA staining in the colons of ND- and HFD-fed mice (Scale bars, 50 μm). (b) Relative Caspase-1 activity in the colons. (c) Serum LDH contents. (d) Serum IL-1β levels. (e) Colonic mRNA expression of pyroptosis-associated genes. (f) Colonic protein expression of pyroptosis-associated genes. (g) Densitometric analyses of Figure 1f. (h) Fecal GSDMD content. For all the panels, n = 5 mice/group. All the values are presented as the mean ± SD. The data were analyzed by using one-way ANOVA followed by Fisher’s LSD post hoc test. *P < .05, **P < .01 compared to the ND group.

### Conventional T lymphocytes and intestinal epithelial cells are the sources of GSDMD-N in the colon of HFD-fed mice

**Figure 2.**
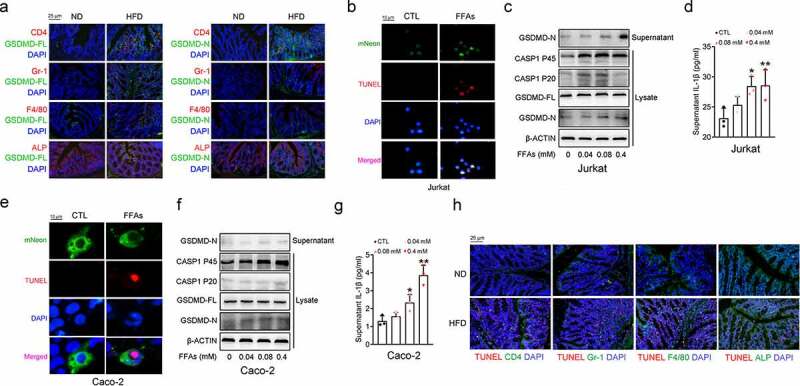
Conventional T Lymphocytes and Intestinal Epithelial Cells are Sources of GSDMD-N in the Colon of HFD-fed Mice

GSDMD is widely distributed in various cell types of the colon, including conventional intestinal epithelial cells and immune cells, such as macrophages, neutrophils, and T lymphocytes.^21^ To investigate the sources of secreted GSDMD-N, we performed IF analysis to identify the source of the increased GSDMD-N levels in response to HFD feeding. As shown in Figure 2(a), the increased protein expression of GSDMD-FL and GSDMD-N in the colons of the HFD-fed mice was mainly localized to CD4-positive T lymphocytes and to ALP-positive enterocytes, and modestly expressed in the Gr-1-positive neutrophils, but rarely accumulated in the F4/80-positive macrophages. To further confirm the sources of HFD-induced colonic GSDMD-N *in vitro*, we treated Jurkat cells (a human T lymphocyte cell line), Caco-2 cells (a colorectal adenocarcinoma-derived cell line), and HL-60 (a human promyeloid leukemia cell, which represents the neutrophils *in vitro*^22^) cells with 0.4 mM free fatty acids (FFAs, an equimolar mixture of oleic acid and palmitic acid) for 24 hours. As shown in Figure 2(b), FFA stimulation increased the green fluorescence aggregation of mNeon-GSDMD (a plasmid constructed to visualize the pyropotsis *in vitro*) in Jurkat cells. Consistently, increased protein expression and secreted levels of GSDMD-N were observed in these FFA-treated Jurkat cells (Figure 2(c,d) and Supplementary Figure 1(a)). Similar results were also observed in FFA-treated Caco-2 and HL-60 cells (Figure 2(e–g). Supplementary Figure 1(b) and Supplementary Figure 2(a–c)). Additionally, it should be noted that the secretion of the active GSDMD-N is closely related to the cell pyroptosis.^23^ Therefore, we evaluated the cell pyroptosis by TUNEL assays,^18^ and found that higher numbers of TUNEL-positive T lymphocytes and enterocytes were detected in the colons of the HFD-fed mice, whereas the Gr-1-positive neutrophils and F4/80-positive macrophages were mildly infiltrated (Figure 2(h)). Hence, although FFAs triggered GSDMD activation in HL-60 cells *in vitro*, neutrophils were not responsible for the HFD-induced colonic GSDMD-N *in vivo*. Collectively, these results indicate that T lymphocytes and enterocytes are dominant producers of the secreted GSDMD-N in the colons of HFD-fed mice.

10-week-old mice were fed either a HFD or a normal diet for 16 weeks (n = 5 mice/group). (a) IF analysis of the colonic colocalization of GSDMD-FL and GSDMD-N with CD4, Gr-1, F4/80 and ALP (Scale bars, 25 μm). (b) IF analysis of the colocalization of mNeon and TUNEL signals in Jurkat cells (Scale bars, 10 μm). (c) Pyroptotic protein levels in the lysates and supernatants of Jurkat cells treated with the indicated doses of FFAs. (d) IL-1β levels in the supernatants from the Jurkat cells. (e) IF analysis of the colocalization of the mNeon and TUNEL signals in Caco-2 cells (Scale bars, 10 μm). (f) Pyroptotic protein levels in the lysates and supernatants of Caco-2 cells treated with the indicated doses of FFAs. (g) IL-1β levels in the supernatants of Caco-2 cells. (h) IF analysis of the colonic colocalization of TUNEL with CD4, Gr-1, F4/80 and ALP (Scale bars, 25 μm). Neon-GSDMD plasmids were transfected into two cell lines for 24 hours, followed by FFAs treatment for another 24 hours. All the values are presented as the mean ± SD. The data were analyzed by using one-way ANOVA followed by Fisher’s LSD post hoc test. *P < .05, **P < .01 compared to the control group.

### GSDMD restrains HFD feeding-induced systemic endotoxemia

**Figure 3.**
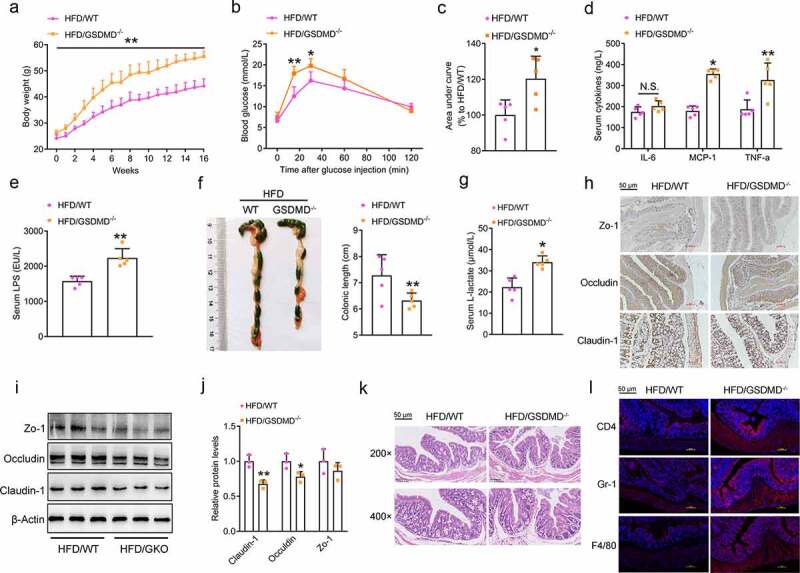
GSDMD Restrains HFD Feeding-induced Systemic Endotoxemia

We next fed GSDMD-deficient mice a HFD for 16 weeks to further dissect the role of colonic GSDMD. As shown in Figure 3(a), the HFD-fed GSDMD^−/−^ mice gained more body weight than the HFD-fed wild-type (WT) mice. Glucose tolerance test (GTT) analysis indicated that these mice displayed severe glucose intolerance (Figure 3(b,c)). Consistently, the serum levels of proinflammatory cytokines (e.g., IL-6, MCP-1 and TNF-α) were increased in these mice (Figure 3(d)). It should be noted that the serum levels of LPS were dramatically increased when GSDMD was knocked out (Figure 3(e)). Collectively, the whole-body GSDMD^−/−^ mice exhibited more severe systemic endotoxemia in response to HFD feeding.

WT and GSDMD^−/-^ mice were fed a HFD for 16 weeks.(a) Body weight. (b) Glucose tolerance test. (c) Area under curve of Figure 3b. (d) Serum levels of proinflammatory cytokines, including IL-6, MCP-1 and TNF-α. (e) Serum levels of LPS. (f) Morphological analysis of the colons. (g) Serum D-lactate levels. (h) IHC analysis of the colonic expression of permeability-associated proteins, including Zo-1, Occludin and Claudin-1 (Scale bars, 50 μm). (i) Protein levels of Zo-1, Occludin, and Claudin-1 in the colons. (j) Densitometric analyses of Figure 3i. (k) H&E staining of the colonic sections (Scale bars, 50 μm). (l) IF analysis of inflammatory markers, including CD4, Gr-1, and F4/80 (Scale bars, 50 μm). For all the panels, n = 5 mice/group. All the values are presented as the mean ± SD. The data were analyzed by using one-way ANOVA followed by Fisher’s LSD post hoc test. *P < .05, **P < .01 compared to the HFD-fed WT group.

Importantly, systemic elevation of LPS in the serum is a leading cause of endotoxemia and is affected by at least three determinants: 1) increased intestinal permeability, which allows more LPS to cross the intestinal barrier and enter the circulation system;^24^ 2) nonresponsive immune cells, which reduce the clearance of LPS;^25^ and 3) gut dysbiosis, which produces damaging LPS.^26^ As shown in Figure 3(f), shorter colons were observed in the HFD-fed GSDMD^−/−^ mice, indicating impaired colonic function. Functionally, the serum levels of D-lactate were significantly increased in the HFD-fed GSDMD^−/−^ mice compared to their HFD-fed WT littermates (Figure 3(g)). Consistently, the colonic expression levels of permeability-associated proteins (Zo-1, Occludin and Claudin) were correspondingly decreased (Figure 3(h–j)). To our surprise, knockdown of GSDMD also modestly affected the FFA/LPS-induced impairment of permeability, as evidenced by unaltered transepithelial electrical resistance (TEER) and permeability-associated protein expression in the GSDMD-knockdown group compared to the control group (Supplementary Figure 3(a–e)). To further dissect the discrepancy of permeability-associated protein expression existed *in vivo* and *in vitro*, a co-culture system was established to mimic the crosstalk between T lymphocytes and enterocytes. As shown in Supplementary Figure 4(a–c), knockdown of *Gsdmd* in Jurkat cells facilitated LPS-induced impairment of gut permeability in Caco-2 cells as evidenced by a more dramatic reduction of TEER and permeability-associated protein expression, because that T lymphocytes are responsible for the LPS clearance *in vivo*.^27^ Considering that GSDMD knockdown in Caco-2 cells modestly affected the LPS-induced impairment of gut permeability, these results indicate that GSDMD is not an essential factor in maintaining the permeability of intestinal epithelial cells, and such an impairment is possibly influenced by increased external inflammatory signals caused by GSDMD deficiency-induced dysfunction of T lymphocytes.

Because of the importance of immune cells in the regulation of inflammation, we next performed H&E and IF analyses and found that GSDMD deficiency exacerbated HFD-induced immune cell infiltration into the mouse colon (Figure 3(k,l)). Among these immune cells, infiltrating T lymphocytes were the most responsive. However, GSDMD deficiency exerts protective effects by decreasing the severity of inflammation in the liver, which seems to contradict our findings. Then, we first identified the colonic sources of systemic proinflammatory cytokines. As shown in Supplementary Figure 5(a), increased levels of proinflammatory cytokines were produced by GSDMD-deficient intestinal epithelial cells but not immune cells in the colon of mice fed a HFD. Accordingly, the TUNEL assay revealed that GSDMD deficiency caused reduced pyroptosis of immune cells (especially T lymphocytes) but had minor effects on intestinal epithelial cells (Supplementary Figure 5(b)). Similarly, the FFA- or LPS-induced secretion of proinflammatory cytokines was decreased in Jurkat cells but was unaltered in Caco-2 cells when GSDMD was knocked down (Supplementary Figure 5(c,d)). These results suggest that intestinal epithelial cells are an unexpected source of inflammation in the colons of HFD-fed GSDMD^−/−^ mice.

### GSDMD deficiency mice exhibit a transmissible dysbiosis

Endogenous bacteria (*Proteobacteria*) are producers of increased serum LPS in HFD-fed mice.^28^ To determine the unique bacterial source of these increased LPS levels, we performed pyrosequencing-based analysis of bacterial 16S rRNA (V4 region) in the feces of WT and GSDMD^−/−^ mice fed with HFD. The detailed sequence data are presented in Supplementary Table 1. It should be noted that the weighted UniFrac-based PCoA revealed a unique clustering of microbiota composition for each group (Supplementary Figure 6(a)), suggesting that GSDMD deficiency altered the overall structure of the gut microbiota in the HFD-fed mice. Fecal microbiota transplantation (FMT) from HFD-fed GSDMD^−/−^ donors to WT recipients was sufficient to increase the permeability impairment and colonic inflammation compared to those in control mice, indicating that gut microbiota partially mediated the effects of GSDMD (Supplementary Figure 6(b–h)). More importantly, Student’s *t* test indicated that *Firmicutes* was the main bacterial species in the feces of the HFD-fed WT group, whereas *Proteobacteria* (*P* < .001) was the main species in the feces of the HFD-fed GSDMD^−/−^ group (Supplementary Figure 6(i)). Taxonomic profiling indicated that GSDMD deficiency increased the HFD-induced accumulation of the *Proteobacteria* phylum in mouse feces by 1.96-fold (*P* = .0003, Supplementary Figure 6(j)).

### GSDMD-N kills Proteobacteria via binding to cardiolipin (CL) in vitro

**Figure 4.**
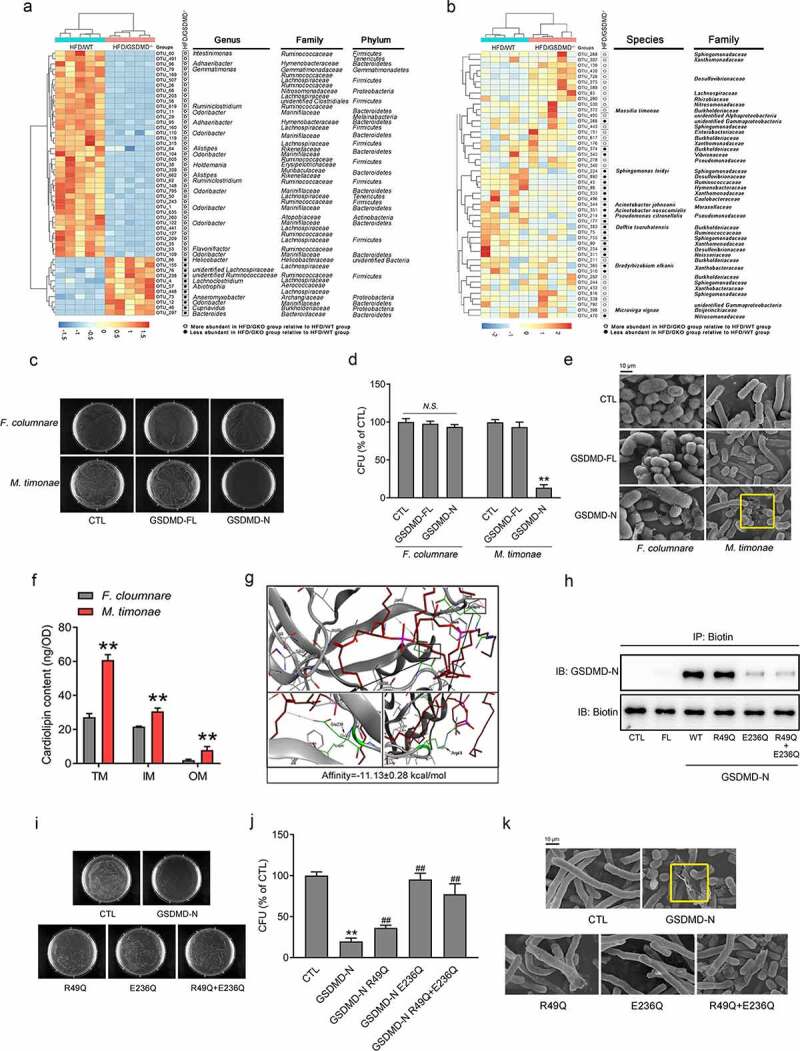
GSDMD-N Kills *Proteobacteria* via Binding to CL *in vitro.*

We subsequently performed clustering analysis to identify the specific bacterial phylum altered by GSDMD deficiency after HFD feeding. Compared to those in the HFD-fed WT group, 50 OTUs were altered in the HFD/GKO group, among which 11 OTUs increased and 39 OTUs decreased (Figure 4(a)). As shown in Figure 4(b), the *Massilia timonae (M. timonae)* was the most responsive bacterium corresponding to the GSDMD deficiency in the feces of HFD-fed mice. In addition, *M. timonae* was also the only *Proteobacteria*, which could be classified to species and purchased commercially. Therefore, we selected it as the representative strain of *Proteobacteria*. Functionally, a bacterial colocalization experiment revealed that *M. timonae* indeed increased serum and fecal LPS levels in mice (Supplementary Figure 7(a–c)), confirming that the *M. timonae* is a representative bacterium for the *Proteobacteria* phylum. To further test our hypothesis, we also selected *Flabobacterium columnar* (*F. columnare*, belongs to *Bacteroidetes*), which was unaltered in response to GSDMD deficiency and was used as a negative control, to investigate the effects of GSDMD on bacterial viability.

(a) Left: Heatmap analysis of the abundance of 50 OTUs that were significantly altered by GSDMD deficiency in the HFD-fed mice based on RDA. Right: Representative bacterial taxa information (genus, family, and phylum) of these OTUs. White or black circles indicate the OTUs that were increased or decreased in the HFD-fed GSDMD^−/-^ group compared to the HFD-fed WT group. OTU taxonomy is shown at right. (b) Left: Heatmap analysis of the abundance of Proteobacteria that were significantly altered by GSDMD deficiency in the HFD-fed mice based on RDA. Right: Representative bacterial taxa information (species and family) of these OTUs. White or black circles indicate the OTUs that were increased or decreased in the HFD-fed GSDMD^−/-^ group compared to the HFD-fed WT group. OTU taxonomy is shown at right. (c) Bacterial colony formation assay. (d) CFUs. (e) Representative scanning electron microscopy images of these bacteria, yellow circle means damaged bacterium (Scale bars, 10 μm). (f) The membrane CL contents of *M. timonae* and *F. columnare*. (g) Molecular docking analysis of GSDMD-N and CL. (h) Immunoprecipitation analysis of the physical interaction between GSDMD-N and biotin-CL. (i) Bacterial colony formation assay. (j) CFUs. (k) Representative scanning electron microscopy images of bacteria treated with the indicated supernatants; yellow circle means damaged bacterium (Scale bars, 10 μm). All the values are presented as the mean ± SD. The data were analyzed by using one-way ANOVA followed by Fisher’s LSD post hoc test. **P < .01 compared to the *F. columnare* group or CTL group, ^##^*P* < .01 compared to the GSDMD-N group, *N.S*. means no significance.

According to previous studies,^20^ we collected and concentrated the culture supernatants from HEK-293 T cells transfected with GSDMD-FL or GSDMD-N plasmids and added these supernatants to bacterial culture media. Real-time detection of the OD_600_ value was used to qualify bacterial biomass and revealed that the growth rate of *M. timonae* was significantly decreased (the final inhibition rate was 56%) in a time-dependent manner when cultured with medium containing the GSDMD-N. In contrast, less powerful inhibitory effects on the growth of *F. columnare* were observed (the final inhibition rate was 30%) (Supplementary Figure 8). Consistently, the bacterial colony formation assay indicated that 24-hour incubation with GSDMD-N-containing medium markedly eliminated the growth of *M. timonae* colonies but modestly affected the numbers of *F. columnare* colonies (Figure 4(c,d)). At the ultrastructural level, scanning electron microscopy analysis showed that treatment with GSDMD-N-containing medium led to a “fried egg” phenotype of the cell wall of *M. timonae*. In contrast, these detrimental alterations almost did not occur in *F. columnare* (Figure 4(e)).

**Figure 5.**
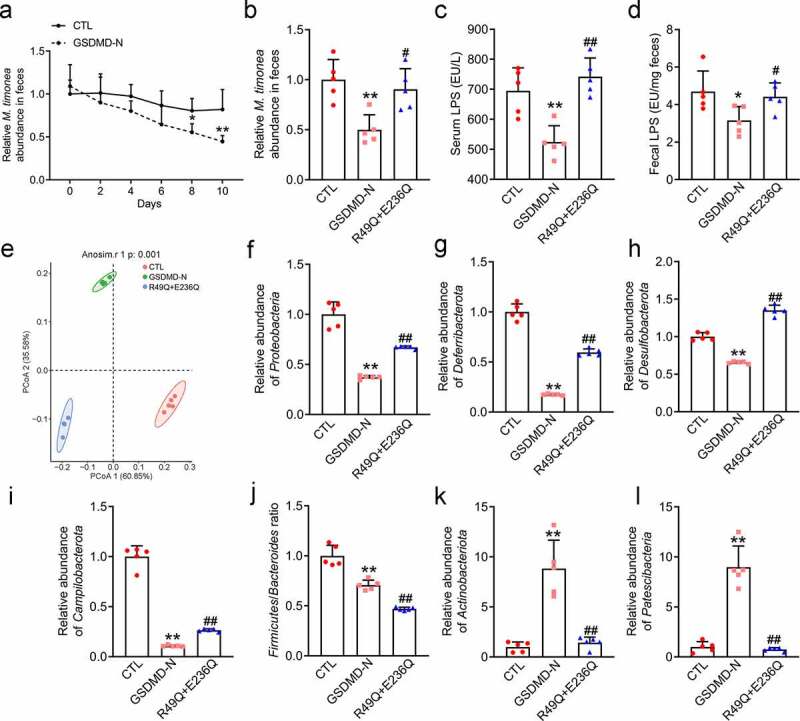
GSDMD-N Kills *Proteobacteria* via Binding to CL in the HFD-fed Mice

Next, we explore the mechanism by which the GSDMD-N protein specifically drives the death of the *Proteobacteria* phylum. It should be noted that GSDMD-N has been demonstrated to kill extracellular bacteria by potentially binding to CL on the cell membrane.^20^ As expected, the content of CL on the cell membrane of *M. timonae* was higher than that on the cell membrane of *F. columnare*, explaining the specific effect of the GSDMD-N proteins on the survival of *Proteobacteria* (Figure 4(f)). However, the direct-binding regions between GSDMD-N and CL remain unknown. To address this issue, we performed molecular docking analysis and found that GSDMD-N exhibited strong CL-binding properties (−11.13 kcal/mol) at the Arg49 and Glu236 sites (Figure 4(g) and Supplementary Figure 9(a)). We next engineered mutant forms of human GSDMD-N containing either Arg49 to Gln or Glu236 to Gln mutations or both. These changes were not predicted to affect the secondary structure of the protein (Supplementary Figure 9(b)), which was verified by using the SOPMA algorithm.^20^ To confirm the essentials of Arg49 and Glu236 sites, we constructed a Biotin-conjugated CL by an esterification reaction (Supplementary Figure 10(a)). The^1^H NMR spectrum of Biotin-CL revealed that peaks at 2.85–3.10 ppm corresponded to the -CH_2_ groups of the biotin, and the peaks at 3.6 ppm could be attributed to the -CH_3_ groups of CL, which indicated the successful conjugation of Biotin-CL (Supplementary Figure 10(b)). Then, we performed immunoprecipitation analysis by using anti-biotin antibody. As shown in Figure 5(h), GSDMD-N WT and CL physically interacted, while the mutation at Arg49 partially blocked such an interaction. More importantly, the mutation at Glu236 dramatically blunted the physical interaction between GSDMD-N and CL. This result further strengthens our conclusion that GSDMD-N exhibits strong CL-binding properties at the Arg49 and Glu236 sites.

(a) Real-time detection of relative *M. timonae* abundance in feces. (b) Relative *M. timonae* abundance in feces at day 10. (c) Serum levels of LPS. (d) Fecal levels of LPS. (e) PCoA plot of weighted UniFrac distance. (f) Relative *Proteobacteria* abundance. (g) Relative *Deferribacterota* abundance. (h) Relative *Desulfobacterota* abundance. (i) Relative *Campilobacterota* abundance. (j) *Firmicutes*/*Bacteroidota* ratio. (k) Relative *Actinobacteriota* abundance. (l) Relative *Patescibacteria* abundance. All the values are presented as the mean ± SD. The data were analyzed by using one-way ANOVA followed by Fisher’s LSD post hoc test. *P < .05 and **P < .01 compared to the CTL group. ^#^*P* < .05 and ^##^*P* < .01 compared to the GSDMD-N group.

Functionally, real-time detection of the OD_600_ value and bacterial colony formation assays revealed that the mutation of GSDMD-N at Glu236 nearly completely abrogated, while the mutation of GSDMD-N at Arg49 partially abolished, the bacterial killing properties of the GSDMD-N proteins (Supplementary Figure 11 and Figure 4(i–k)). Additionally, the results from colony formation assay using the concentrated supernatants were confirmed by using purified proteins of GSDMD-N WT and its mutants (Supplementary Figure 12(a,b)).

### GSDMD-N kills Proteobacteria via binding to CL in the HFD-fed mice

To further verify the anti-bacterial function of these purified proteins in the mouse colon, we purified GSDMD-N WT and its mutant proteins by using affinity chromatography. Note that to avoid the influences of abundant gastrointestinal proteinase and peptidase, we directly injected these purified proteins to the colons of HFD-fed mice according to a previous study.^29^ Because *M. timonae* is the corresponding bacteria of GSDMD-N proteins, we firstly examined the abundance of *M. timonae* every 2 days. We found that the abundance of *M. timonae* from the feces of HFD-fed mice treated with GSDMD-N WT were significantly decreased to 41% at day 10, when compared to the control group (Figure 5(a,b)). Consistently, the serum and fecal levels of LPS were decreased in the HFD-fed mice treated with GSDMD-N WT (Figure 5(c,d)). These data suggest that GSDMD-N WT protein is functional in the colons of HFD-fed mice. In contrast, a double mutation of GSDMD-N purified protein partially abolished the bacterial killing effects of purified GSDMD-N WT on the *M. timonae*, and restored the serum and fecal levels of LPS by 41.5% and 32.5%, respectively (Figure 5(c,d)).

To further assess the effects of GSDMD-N WT and mutants on the composition of gut microbiota *in vivo*, we performed 16s rRNA analysis and found that a unique clustering of microbiota composition for each group (Figure 5(e)). Moreover, injection of GSDMD-N WT proteins similarly decreased the abundance of *Proteobacteria*, while a double mutation of GSDMD-N purified protein partially abolished its bacterial killing properties (Figure 5(f)). These results confirmed the specific effects of host GSDMD-N on endogenous *Proteobacteria*. Interestingly, we also noticed that GSDMD-N WT proteins decreased the abundance of other bacteria, including *Deferribacterota, Desulfobacterota* and *Campilobacterota* (Figure 5(g–i)). Moreover, the *Firmicutes*/*Bacteroides* ratio was decreased (Figure 5(j)), while the abundance of *Bacteroides* was increased by 45.1% in response to the GSDMD-N WT protein, suggesting the specific bacterial killing properties of GSDMD-N targeting on the detrimental bacteria. Similarly, the double mutation (R49Q+E236Q) partially abrogated the bacterial killing properties of the GSDMD-N WT protein (Figure 5(f–j)), further confirming the Arg49 and Glu236 are essential sites for the anti-bacterial function of GSDMD-N. Besides, we also found that the abundances of *Actinobacteriota* and *Patescibacteria* were increased by the GSDMD-N WT protein, whereas was decreased in the double mutant protein-treated group (Figure 5(k,l)).

## Discussion

In this study, we identified the precise role of colonic GSDMD in antagonizing the HFD-induced systemic endotoxemia, colonic inflammation, and impaired intestinal barrier, as well as gut dysbiosis. We found that HFD feeding induced the expression and secretion of GSDMD-N protein in both CD4-positive T lymphocytes and enterocytes within the mouse colon. More importantly, GSDMD deficiency reduced inflammatory responses of T lymphocytes corresponding to HFD feeding, while modestly affected the HFD-induced inflammation in the enterocyte. These results indicate that colonic GSDMD is a *per se* critical determinant of the immune cells rather than the enterocytes. On the other hand, we specifically dissected the *Proteobacteria* phylum as the bacterial target for the host GSDMD-N and authenticated the functional interaction between host GSDMD-N and bacterial membrane phospholipid CL at the Arg49 and Glu236 sites. Although the potential interaction has been achieved before,^30^ we further compensated the key sites for such an interaction. Notably, because of cleavage by Caspase-1, the Glu236 site of GSDMD was exposed to the external environment, thus leading to an increased potential to interact with other factors, including CL. Therefore, our findings demonstrate that this site is not only a key residue for protein cleavage but also an important site for the bacterial recognition of GSDMD, which explains the inability of full-length GSDMD to kill bacteria.

HFD feeding has been documented to activate GSDMD-N expression and pyroptosis in various mouse tissues, including liver, adipose tissue and kidney.^17,18,31^ However, the direct factors which delivers the HFD signals to the GSDMD are incompletely identified. Of note, LPS and inflammatory Caspases (e.g., Caspase-1/11 or Caspase-4/5) are essential factors to induce the cleavage of the GSDMD-N domain and further triggering the cell pyroptosis.^32^ More importantly, these two factors are often increased by HFD feeding signals in mice, hence providing a prerequisite for the GSDMD activation and subsequent pyroptosis.^16^ In the present study, we also confirmed that the expression and activity of inflammatory Caspase-1 were elevated in the colons of HFD-fed mice, further demonstrating the critical role of Caspase-1 in mediating HFD signals to GSDMD activation. On the other hand, we also noticed the FFAs also triggered the GSDMD activation and pyroptosis in both immune cells (Jurkat and HL-60 cells) and enterocytes, suggesting FFAs are another potential factor mediating the HFD signals, and thus activating the GSDMD and cell pyroptosis. Further investigations may focus to identify the detailed types of lipids which triggers GSDMD activation by using lipids with different carbon–oxygen ratio in the immune cells. Besides, when suffering from the HFD signals, the white adipose tissue is a critical organ to secrete inflammatory factors, including TNF-α, MCP-1 and IL-6, to the blood circulation. These inflammatory cytokines can be effectively taken by the immune cells and enterocytes in the colons,^33^ which further potentially activating the colonic GSDMD cleavage and pyroptosis, and finally impairing the gut permeability and inducing the gut dysbiosis.

Over the five years, the metabolic regulatory roles of GSDMD have been gradually documented. For instance, HFD or methionine- and choline-deficient diet triggers the pyroptosis and GSDMD cleavage in the mouse liver, while loss-of-function of GSDMD prevents mice from nonalcoholic fatty liver disease and nonalcoholic steatohepatitis progression.^17^ In addition, adipose GSDMD serves as the target protein of melatonin, a well-known anti-obesity hormone.^18^ These global GSDMD-deficient *in vivo* studies imply the detrimental roles of GSDMD in metabolic diseases. However, in our study, colon-derived GSDMD-N exhibited protective effects on the HFD-induced metabolic endotoxemia and gut dysbiosis. These contradictory results may be explained by the different origins from either immune or nonimmune cells, which has been observed in many other studies. For example, the cardiomyocyte-activation of GSDMD induces the systemic inflammation,^16^ whereas the neutrophil-activation of GSDMD exerts anti-inflammatory effects.^34^ Interestingly, we found that the HFD-induced colonic GSDMD-N are secreted by both CD4-expressing T lymphocytes and ALP-expressing enterocytes. Functionally, GSDMD deficiency reduced the immune responses and decreased the LPS/FFA-triggered cell death within the T lymphocytes, rather than the enterocytes, indicating that host GSDMD mainly exerts its pyroptotic function in immune cells in the colon of mice suffering from the chronic HFD feeding. More importantly, while knockdown of GSDMD in Caco-2 cells exhibited modest impacts on the LPS/FFA-disrupted gut permeability. Hence, the potential function of GSDMD involved in the enterocyte need to be further investigated by using enterocyte-specific GSDMD-deficient mice.

GSDMD is identified in the mouse immortalized bone-marrow-derived macrophages for its pro-pyroptotic function,^19^ indicating that GSDMD is a critical factor in maintaining the immune cell function. Consistently, the immune cell infiltration is reduced in the liver of either HFD-fed or MCD-fed GSDMD^−/-^ mice, which differs from our results in the colon.^17^ This discrepancy is due to different immune cell types corresponding to HFD feeding in the liver and colon. Briefly, macrophages are the most infiltrated cells in the steatotic liver,^35^ while the T lymphocytes are the dominant immune cell types in the colon of HFD-fed mice.^36^ In addition, although colonic proinflammatory macrophages mediate the HFD-induced insulin resistance and systemic inflammation,^37^ our findings provided strong evidence that HFD feeding triggered the accumulation and pyroptosis of T lymphocytes, rather than the macrophages and neutrophils, as evidenced by TUNEL analysis. Given that the gut microbiota regulates intestinal CD4-positive T cells to control metabolic diseases,^38^ the HFD-induced colonic macrophage activation may be the secondary effects caused by the T cell pyroptosis and its-related gut dysbiosis. Of note, the influences of GSDMD on the T lymphocyte function are tissue-specific. For example, T cell infiltration is reduced in the CNS, whereas increases in the spleen of the GSDMD^−/-^ EAE mice.^39^ In the colon, increased the CD4-positive T cells are detected when the GSDMD is deficient in the DSS-treated colitis mice,^19^ which is similar to our findings that GSDMD deficiency increased T cell infiltration in the colon of HFD-fed mice, even these cells were nonfunctional and ineffective in response to HFD/LPS. Because the diverse types of T cells exist, further studies are encouraged to dissect the cell type-specific role of GSDMD within the T lymphocytes.

GSDMD is known to exert anti-bacterial function in protecting hosts from excessive infiltration of exogenous gram-negative bacteria, including *E. coli, S. typhimurium*, and *B. cenocepacia*.^16,20,21^ However, the effects of GSDMD on the endogenous bacteria remain elusive. In the present study, we found that GSDMD deficiency increased the HFD-induced accumulation of the *Proteobacteria* phylum, suggesting that *Proteobacteria* serves as a potential bacterial target of GSDMD-N. On the other hand, GSDMD deficiency modestly affects DSS-disrupted gut microbiota and exacerbates DSS-induced colitis in a microbiota-independent manner.^19^ Such a discrepancy may be caused by the different inflammatory settings. For the HFD feeding, alterations of gut microbiota composition, including the increased ratio of *Firmicutes* to *Bacteroidetes*, and accumulation of *Proteobacteria* phylum, are caused by both diet and host cell-secreted proteins, thus leading to a low-level and chronic inflammatory status.^38,40^ In contrast, for the DSS treatment, changes of bacterial composition, including the accumulation of *Firmicutes, Bacteroidetes*, and *Proteobacteria* phyla, are induced by great impacts of drug toxicity on the colon system.^41^ At the meanwhile, although DSS dramatically induces the GSDMD-N production in colonic macrophages, the protein amount is not capable to antagonize such a drug-induced influence.^19^ Hence, we believed that HFD-fed animal model in this work is more appropriate to demonstrate the *per se* endogenous targeted bacteria of GSDMD in the colon. However, due to the commercial limitation of bacterial resources, we are not able to confirm the specificity of GSDMD-N by using more bacteria. Fortunately, we confirmed the interaction and identified the direct binding sites between GSDMD and CL, which will help to dissect more GSDMD-target bacteria.

*Deferribacterota, Desulfobacterota* and *Campilobacterota* are demonstrated to be involved in the activation of systemic inflammation.^42^ In the present study, by directly injecting the purified GSDMD-N WT protein into the mouse colon, we found that except for the *Proteobacteria*, GSDMD-N also triggered the death of aforementioned three bacterial phyla. Given the detrimental effects of these bacteria on the intestinal microenvironment, our results suggest that GSDMD-N is a beneficial protein in maintaining the homeostasis of gut microbiota under HFD feeding conditions. Interestingly, we also notice that another type of detrimental bacterial phylum,^43^ *Actinobacteriota*, was increased corresponding to the injection of the GSDMD-N WT protein. Besides, a newly identified bacterial phylum, *Patescibacteria*, exhibited a similar tendency. However, the detailed mechanism of GSDMD-N specific killing *Proteobacteria, Deferribacterota, Desulfobacterota*, and *Campilobacterota*, but not other bacteria, remain elusive. Notably, CL is a well-known lipid enriched on various bacterial membranes,^44^ and possesses protein binding abilities with GSDMD-N.^32^ In the present study, we found that CL contents were abundantly expressed on the membrane of *Proteobacteria*, when compared to *Bacteroidetes*. More importantly, GSDMD-N recognized and physically interacted with CL at its Arg49 and Glu236 sites. Hence, the abundance of CL on the bacterial membranes may be a determinant of the GSDMD-N-specific killing of bacteria, and a further detection of membrane CL contents on the detailed species of aforementioned bacteria could explain such a specificity. Last but not the least, since we identified only *Proteobacteria* are the target bacteria of GSDMD-N in the colons of HFD-fed GSDMD^−/-^ mice, the purified protein-induced discrepancy may be possible explained by the reason that the artificial injection into the colon leads to an increased local concentration of the GSDMD-N protein and raises the contact possibility with bacteria, thus causing a more direct impact on the composition of gut microbiota.

**Figure 6.**
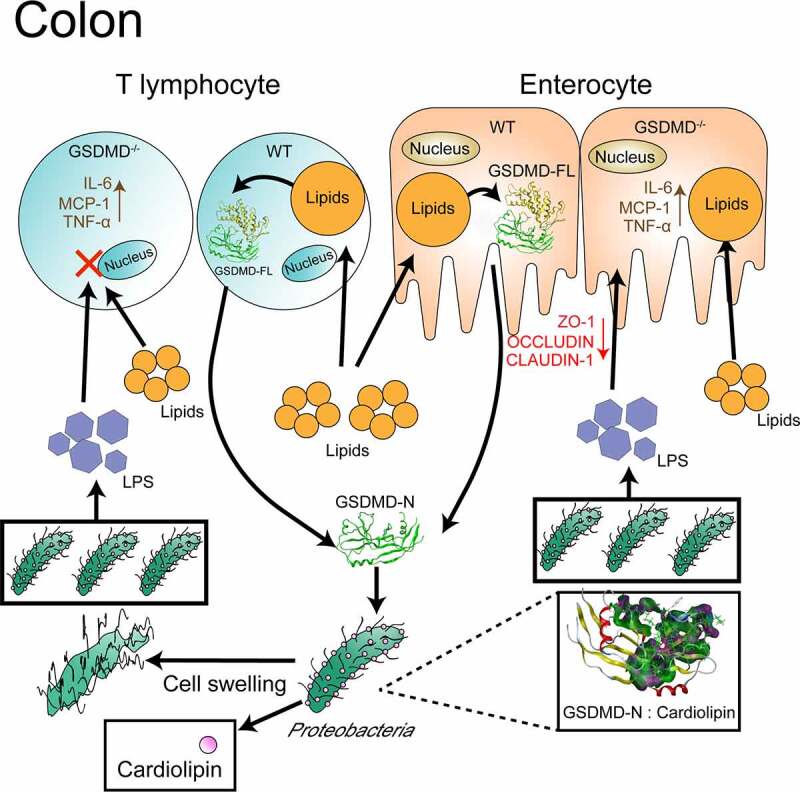
The mechanism by which CD4-positive T lymphocyte- and enterocyte-secreted GSDMD-N maintains low levels of HFD-induced metabolic endotoxemia by inhibiting *Proteobacterial* growth

In this study, we focused on the role of GSDMD in the regulation of HFD-induced systemic endotoxemia. Our findings explain the mechanism by which CD4-positive T lymphocyte- and enterocyte-secreted GSDMD-N maintains low levels of HFD-induced metabolic endotoxemia by inhibiting *Proteobacterial* growth (Figure 6). Considering the pore forming cytosolic proteins are extremely hazardous to the host cell, oral, and colon-specific delivery of GSDMD-N mimetic in accurate doses could be an attractive strategy for the treatment of HFD-induced metabolic endotoxemia.

## Materials and methods

### Bacteria

*M. timonae* (#CGMCC 1.12368) and *F. columnare* (#CGMCC 1.12742) were purchased from the China General Microbiological Culture Collection Center (CGMCC). Both bacterial strains were cultured in R2A medium at 30°C.

### Mice

GSDMD^−/−^ mice on a C57BL/6 J background were kindly provided by Professor Feng Shao (National Institute of Biological Sciences, Beijing, China). All the animal procedures in this investigation conformed to the Guide for the Care and Use of Laboratory Animals published by the US National Institutes of Health (NIH publication No. 85–23, revised 1996) and the approved regulations established by the Laboratory Animal Care Committee at China Pharmaceutical University (Permit number SYXK-2016-0011). The mice were maintained in a temperature- and humidity-controlled environment with a 12-h light-dark cycle. For the HFD feeding experiments, 10-week-old males were randomly chosen to receive either a HFD (60% kcal from fat, Research Diets, New Brunswick, NJ, USA) or ND diet for 16 weeks.

For fecal transplantation,^45^ all the procedures were performed according to a previously described protocol. Briefly, donor mice (n = 5 mice/group) were selected either from the HFD-fed WT group or the GSDMD^−/−^ group as described above. After 8 weeks of HFD feeding, stools were collected daily for the subsequent 8 weeks. The stools from the donor mice of each group were pooled, and 100 mg was resuspended in 1 mL of sterile saline. The solution was vigorously mixed for 10 s by vortexing before centrifugation at 800 × g for 3 min. The supernatant was collected and used as the transplant material. Fresh transplant material was prepared on the day of transplantation within 10 min before oral gavage to prevent changes in the bacterial composition. Eight-week male recipient mice (n = 5 per transplant group) were inoculated daily with fresh transplant material (100 mL for each mouse) by oral gavage for 8 weeks before being humanely sacrificed.

For bacterial colocalization experiment, 8-week-old WT mice were received a mixture of neomycin, metronidazole, and ampicillin by gavage at the dose of 200 mg/kg body weight for consecutive 5 days. Then, these mice were gavaged with either killed or alive *M. timonae* at the concentration of 10^8^ CFU/200 μL for 8 weeks. Equal volume of sterile saline was used as the control group.

### Cell culture

The human HEK-293 T, Caco-2, Jurkat and HL-60 cell lines were purchased from American Type Culture Collection. For the HEK 293 T cell line, the cells were grown routinely in DMEM (Gibco, #8119007, Waltham, USA) supplemented with 10% *v*/*v* heat-inactivated fetal bovine serum (FBS, Gibco #15140122, Waltham, USA) and 100 U/mL PenStrep at 37°C and 5% CO_2_. For the Caco-2 cell line, the cells were cultured in DMEM supplemented with 10% FBS, 1% nonessential amino acids (Gibco #11140-050, Waltham, USA) and 100 U/mL PenStrep at 37°C and 5% CO_2_. For the Jurkat and HL-60 cell line, the cells were cultured in RPMI 1640 supplemented with 10% FBS, 1% sodium pyruvate and 100 U/mL PenStrep at 37°C and 5% CO_2_.

### Serological analysis

Blood samples were collected in nonheparinized tubes and centrifuged at 4000 rpm for 10 min at 4°C. The serum levels of molecules, including TNF-α, MCP-1, IL-6, D-lactate and LPS, were determined spectrophotometrically using commercial kits (Ruixin Biotechnology Co., Ltd. Quanzhou, China).

### Colonic histology

Colons were isolated, fixed in a 4% paraformaldehyde solution for 24 hours *in situ*, processed for paraffin embedding, and then cut into 6-μm transverse sections for routine hematoxylin and eosin (H&E) staining.

### FLICA staining

FAM-FLICA Caspase-1 assays were performed by using a commercial kit (ImmunoChemistry Technologies, Bloomington, USA). Briefly, slides were fixed with 1× fixative for 30 min and then washed with 1× apoptosis wash buffer (AWB) for 15 min. Each vial of FLICA was reconstituted with 50 μL DMSO to prepare a 150× stock. Then, FLICA was immediately diluted at a ratio of 1:5 by adding 200 μL PBS to each vial to prepare the 30× FLICA solution. Finally, the sections were incubated with 1× FLICA solution at room temperature (RT) for 4 hours, after washing, the sections were incubated with 1 × PI solution for 5 min in the dark at RT.

### Caspase-1 activity analysis

Caspase-1 activity was determined using a specific assay kit based on the catalytic function of activated caspase-1 on the substrate Ac-YVAD-pNA (Beyotime Technology, Nantong, China) following the manufacturer’s protocol. Absorbance values were considered equivalent to caspase-1 activity units and adjusted based on the total amount of protein per sample.

### TUNEL assay

Cell death was detected by TUNEL staining with a TUNEL Apoptosis Assay Kit (Vazyme, Nanjing, China). The cell nuclei were stained with DAPI (5 μg/mL, Beyotime Technology, Nanjing, China) after TUNEL staining.

### Synthesis and characterization of the Biotin-CL conjugates

The synthetic procedure for the Biotin-CL was adapted from a previously reported method with minor modifications.^46^ Briefly, 3.2 mg biotin, 9.2 mg NHS and 1.536 mg EDC were added to 1.5 mL ddH_2_O. Then, 10 mg of CL (sodium salt) diluted in 200 μL ethanol was added to the mixture, and allowed to react for 24 h at room temperature. Subsequently, the solution was dialyzed against distilled water for 3 days and lyophilized to obtain the pure Biotin-CL product according to a previously reported method. Characterization of the Biotin-CL was performed using ^1^H NMR (AVANOE, Bruker, Rheinstetten, Germany).

### Immunoblotting

Proteins were extracted by using RIPA buffer, and protein expression levels were examined by Western blot as described previously. It should be noted that for the fecal proteins, approximately 100 mg of feces was collected and lysed with RIPA lysis buffer (in a water bath at 37°C for 30 min). After electrophoresis, the proteins were transferred onto nitrocellulose membranes, which were incubated with primary antibodies, followed by incubation with HRP-conjugated secondary antibodies. The immunodetected proteins were visualized using an ECL assay kit (Millipore, Burlington, USA). The antibody against β-actin (Cat#: GB11001) was obtained from Servicebio (Wuhan, China; 1:2000 dilution). The antibody against GSDMD (#sc81868) was purchased from Santa Cruz (Dallas, USA; 1:500 dilution). The antibodies against IL-1β (#A1112) and NLRP3 (#A5652) were purchased from ABclonal (Cambridge, USA; 1:1000 dilution). The antibodies against Caspase-1 (#22915-1-AP), ZO-1 (#21773-1-AP), Occludin (#27260-1-AP) and Claudin-1 (#13050-1-AP) were obtained from Proteintech (Chicago, USA; 1:1000 dilution).

### RT-qPCR assay

Total RNA was extracted from colonic tissues using TRIzol® Reagent (Ambion, Waltham, USA), reverse-transcribed to complementary DNA using a High-Capacity cDNA Reverse Transcription Kit (Takara, Kyoto, Japan), and analyzed by qPCR using SYBR Green (Vazyme, Nanjing, China) and the LightCycler^@^ 480 System (Roche, Basal, Switzerland). All the primer sequences are listed in Supplementary Table 2. The relative abundance of the target genes was normalized to the abundance of *β-Actin*.

For the quantification of *M. timonae*, RT-qPCR system was as follows: qPCR SYBR Mix 5 μL, primers (20 μM) 0.4 μL, DNA 20 ng, with double-distilled water to 10 μL. The PCR program was as follows: 95°C for 5 min; 95°C for 10 s, 60°C for 30 s, 40 cycles; 95°C for 10 s, 65°C for 1 min; the fluorescence signal was detected at the end of each cycle extension step. The primer sequences for the *M. timonae* are listed in Table S2.

### Fecal DNA extraction and pyrosequencing

Stool samples were collected from each mouse in the 8^th^ week and immediately snap frozen in liquid nitrogen before storage at −80°C. The feces were collected after administration and extracted using a fecal DNA isolation kit (Qiagen, Germantown, USA). The extracted DNA from each sample was used as the template to amplify the V4 region of the 16S rRNA genes for subsequent pyrosequencing, which was performed by Novogene (Beijing, China) as described previously.^47^

### Immunoprecipitation

Fifty μg of the indicated purified protein was reacted with 100 mg Biotin-CL overnight at 4°C, the mixture was incubated with 20 μL of protein-A/G agarose beads and 2 μg of anti-Biotin (#sc81868, Santa Cruz, Dallas, USA; 1:500 dilution) in the following day. After 24-h incubation, the immune complexes were centrifuged and washed 5 times with ice-cold washing buffer. The immunoprecipitated protein was further analyzed using Western blot analysis.

### IF and IHC analyses

For immunofluorescence, frozen colons, fixed mouse primary adipocytes or frozen livers were cut into 6-μm transverse sections. The sections were then treated overnight at 4°C with a negative control reagent (5% goat serum, AR0006, Boster, Wuhan, China) or diluted primary antibodies. For immunohistochemistry, fresh colonic samples were fixed in 4% paraformaldehyde solution for 24 h *in situ*, processed for paraffin embedding, and cut into 6-μm transverse sections for routine IHC staining. The slides were incubated with antibodies at 4°C overnight for subsequent immunostaining by using diaminobenzidine (DAB). The antibodies against GSDMD (#sc81868 for GSDMD-FL and #sc393581 for GSDMD-N) were purchased from Santa Cruz (Dallas, USA; 1:100 dilution). The antibodies against ZO-1 (#21773-1-AP), Occludin (#27260-1-AP), and Claudin-1 (#13050-1-AP) were obtained from Proteintech (Chicago, USA; 1:200 dilution). The antibodies against F4/80 (#GB11027), GR-1 (#GB11229), IL-6 (#GB11117), TNF-α (#GB11188) and MCP-1 (#GB11199) were purchased from Servicebio (Wuhan, China; 1:200 dilution). The antibody against CD4 (#XS20200521010) was purchased from Bioworld (Nanjing, China; 1:200 dilution). Next, the slides were incubated with anti-mouse and anti-rabbit immunofluorescent (Alexa Fluor 555/488 Conjugate) secondary antibodies (Cell Signaling Technology, Danvers, USA). The sections were photographed with a Nikon microscope (ECLIPSE, Ts2R-FL, Tokyo, Japan).

### Co-culture experiments

To mimic the crosstalk between T lymphocytes and enterocytes, we performed the co-culture experiments. Cells were cultured using cell culture inserts (50 mm pore size) to separate both cell populations. Caco-2 cells were seeded on the bottom of the plate, while the Jurkat cells were plated on the insert. siRNA targeted *Gsdmd* gene was transfected into Jurkat cells. After 24-h co-culture, LPS was incubated in Jurkat cells for another 24 h. Finally, TEER of Caco-2 monolayer was measured, the expression levels of permeability-associated proteins were also detected by Western blot analysis.

### Transfection and supernatant collection

Cell transfection was performed using Lipofectamine 3000 transfection reagent (Invitrogen, USA) according to the manufacturer’s instructions. To collect the N-GSDMD-enriched cell supernatant, 5.5 µg of the indicated plasmids was transfected into HEK-293 T cells cultured in 10-cm dishes without antibiotics. Forty-eight hours after transfection, the supernatants were collected and concentrated five times by using a lyophilizer .

### Bacterial growth assay

CFU assays and turbidimetry were used to measure bacterial growth as previously described.^20^ Briefly, for turbidimetry, bacteria were diluted (1:1000) in bacterial culture medium following treatment and incubated with successive shaking at 30°C in a 200 μL volume. Growth curves were monitored by reading absorbance at 600 nm over 16 h using a The Spark® Multimode Microplate Reader (Tecan, Switzerland).

### Molecular docking

To explore the binding mode of N-GSDMD with bacterial cell wall components, molecular docking calculations were carried out using Molecular Operating Environment (MOE) 2015.10 (Molecular Operating Environment Software, US). During molecular docking, several steps, such as removing water molecules and adding hydrogens, were required. GSDMD-N was used as the receptor, and CL was used as the ligand.

### Extraction of total membrane lipids

*M. timonae* and *F. columnare* strains were grown to the exponential phase in 100 mL R2A medium, and the cell suspensions were harvested by centrifugation (3000 rpm, 10 min, 4°C). The pellets were washed twice in PBS (10 mL) and then extracted. The total lipid fraction (TL) was extracted in 20 volumes of chloroform/methanol (2:1, *v*/*v*) and chloroform/methanol/water (CMW; 1:2:0.8, *v*/*v*). After removing the insoluble reagents, the extracts were dried under nitrogen and subjected to biphasic partitioning in 1-butanol and water (2:1, *v*/*v*). The organic phase was dried, and the lipids were resuspended in 40 µL/100 mg (pellet wet weight) water-saturated 1-butanol. The remaining lipids (IM lipids) were extracted in water-saturated 1-butanol (RT for 30 min) to selectively remove the OM lipids. After centrifugation (1500 rpm, 5 min, 4°C), the pellets were sequentially extracted in 20 volumes of chloroform/methanol (2:1, *v*/*v*) and then CMV (1:2:0.8, *v*/*v*). Membrane lipid-CL was determined spectrophotometrically using commercial kits (XinYu Biotechnology, Shanghai, China).

### Scanning electron microscopic analysis of bacteria

Bacteria were collected by centrifugation (3000 rpm, 10 min, 4°C), fixed with 2.5% glutaraldehyde overnight, and then rinsed with PBS three times. The samples were dehydrated with a graded series of ethanol (30, 50, 70, 95, and 100%) and dried by the tertiary butanol method. The dried specimens were sputter coated with gold-palladium and imaged with a Hitachi Regulus 8100 (Tokyo, Japan) operating at 3 kV.

### Protein purification

6× His-tagged recombinant plasmids of GSDMD-FL, GSDMD-N (WT, R49Q, E236Q, and R49Q+E236Q) were transfected into HEK-293 T cells. After 48 h, cells were collected and disrupted by non-denaturing lysis buffer with 0.05% Tween 20, and then were purified using BeyoGold^TM^ His-tag Purification Resin (Beyotime Technology, Nantong, China).

### Colorectal injection of purified proteins

The purified GSDMD-N WT and its double mutant (R49Q+E236Q) proteins were given to 4-month HFD-fed mice by colorectal injection every 2 days. Briefly, after 24-h food deprivation, mice were anesthetized by continuously isoflurane (2–3% for induction and 1.5–2% for maintenance), and their distal colons were carefully cleaned with small balloon catheters. Then, the purified proteins (dissolved in saline, 12.5 mg/kg body) were injected into the colons. This solution (freshly prepared on the treatment day) was injected (0.2 mL) into the colon 3 cm proximal to the anus, using a cannula left in place for 1 min to ensure that the solution was not immediately expelled by the mice. The mice were then maintained in a head-down position for 30 min. After 10 days, mice were sacrificed and feces were collected for 16S rRNA (V4) pyrosequencing by Novogene (Beijing, China).

### Statistical analyses

Descriptive statistics were performed with were performed with GraphPad Prism 7.0 (GraphPad Software, US). Groups of data are presented as the mean ± standard deviation (SD). Data were analyzed by using one-way ANOVA followed by Fisher’s LSD post hoc test.

## Author contributions

Yujie Shi, Yixin Zou, Yonghong Xiong, Shiyao Zhang, Mingming Song conducted researches. Wenxiang Zhang, Xiaofei An and Chang Liu provided advices. Yujie Shi, Siyu Chen edited the paper. Yujie Shi, Yixin Zou, Yonghong Xiong, Wenxiang Zhang, Siyu Chen conceived the study, designed experiments, and wrote the paper.

## Disclosure of potential conflicts of interest

No potential conflict of interest was reported by the author(s).

## Supplementary Material

Supplemental Material

## Data Availability

Data used in this study are available from the authors upon reasonable request.
